# Household Hints and Recipes

**Published:** 1873-07

**Authors:** 


### Household Hints & Recipes.

To Clean Kid Gloves.—Take one part of curd soap and three parts of water. Mix with heat, and then stir in one part of essence of citron. A little of this rubbed over the kid will readily remove all dirt without injuring the glove or leaving any unpleasant odor.

To Remove Paint and Putty from Window Glass.—Put sufficient saleratus into hot water to make a strong solution, and with this saturate the paint which adheres to the glass. Let it remain until nearly dry, then rub it off with a woolen cloth.

Tobacco Chewers.—Of the many chewers of tobacco in these days, there must be many who wish to relinquish the habit. It is said that a little coarsely cut gentian root, well masticated, (the saliva being swallowed), taken after every meal, will soon take away all desire for the chewing of tobacco.

Raspberry Jam.—Let the raspberries be thoroughly ripe. Mash them with a wooden spoon. To every pound of raspberries add a pound of sifted sugar. Boil well half an hour, stirring it continually, lest it should burn.—When of a good thickness, put it into pots, let it cool thoroughly, and cover with brandied paper.

To Render Cloth and other Fabrics Moth and Water-proof.—A solution of acetate of alumina is prepared by mixing solutions of equal weights of alum and sugar of lead. The clear liquid is diluted and mixed with a solution of isinglass. In this mixture the articles are left for about twelve hours, until they are thoroughly saturated, when they are dried and pressed, or otherwise finished.—*Farber Ztg.*

Lime for Poisoning by Plants and Insects.—A standing antidote for poison by oak, ivy, etc., is to take a handful of quick-lime, dissolve in water, let it stand half an hour, then paint the poisoned parts with it. Three of four applications will never fail to cure the most aggravated cases. Poison from bees, hornets, spider bites etc., is instantly arrested by the application of equal parts of common salt and bicarbonate of soda, well rubbed in on the place bitten or stung.

Potato Puffs.—The following is from a late English journal: "Take cold roast meat—beef or mutton, or veal and ham together—clear from gristle, cut small, and season with pepper and salt and cut pickles (if liked); boil and mash some potatoes, make them into a paste with an egg, and roll out, dredging with flour. Cut round with a saucer, put some of the seasoned meat on one half, and fold it over like a puff; pinch or nick it neatly round and fry it a light brown. This is a good way to cook meat which has been dressed before.

To Keep Flies from Horses.—One of the simplest means of keeping flies from annoying horses or cattle, is to take a bunch of smartweed, bruise it so as to make the juice exude, and rub the animal thoroughly with this bunch of bruised weed—especially upon his neck, legs, and ears. Neither flies nor other insects will trouble him for at least twenty-four hours. If preferred, an infusion may be made by steeping the weed, and the liquid applied with a sponge.

Blackberry Jam.—Gather the fruit when perfectly ripe, and in very dry weather. Put the blackberries into a jar and place the jar in hot water, keeping it boiling until the juice is extracted from the fruit. Pass it through a fine sieve or jelly-bag without much pressure. For every pint of juice add fourteen ounces of sugar, and boil in a clean preserving-pan about five and twenty minutes, carefully taking off the scum as it rises to the surface. Place it hot in small jars and cover it with thin tissue-paper, dipped in brandy, and brown paper over it. Keep it in a cool, dry place.

The Teeth.—In infancy, the mother should make it a part of the daily care of the child to secure the perfect cleanliness of its teeth. Becoming thus accustomed to it, the child, when old enough to use the brush, will find it impossible to feel comfortable after a meal until the teeth have been cleansed as carefully as the face and hands. Small, soft tooth-brushes and pleasant dentifrices, are now manufactured exactly suited for the purpose; these, if the habit of use is thus early formed, will soon become a necessity to the child's comfort; and the practice thus commenced will be almost sure to be continued through life.

Sweeping Carpets.—Persons who are accustomed to use tea-leaves for sweeping carpets, and find that they leave stains, will do well to employ fresh-cut grass instead. It is better than tea-leaves for preventing dust, and gives the carpet a very bright fresh look.

---

To Remove Ink from Paper.—Shake well together one pound of chloride of lime in four quarts of soft water. Then let it stand for twenty-four hours, after which strain through a clean cotton cloth, and add one tea-spoonful of acetic acid to every ounce of the chloride of lime water. Apply this fluid to the blot, and the ink will disappear. Absorb the fluid with a blotter.

---

Horseradish.—A correspondent of *The Garden* (London) says: "It may not be generally known that if leaves or litter be placed on the tops of horseradish crowns two feet or so thick, the plants grow through them in the course of the summer, making small white roots the thickness of one's finger, which are as tender as spring radishes, and much to be preferred to the tough, stringy stuff usually supplied with our roast beef."

---

Liquid Glue.—An excellent liquid glue can be made by dissolving glue in nitric ether. This ether only takes up a certain quantity of glue, so that there is no danger of the solution being too concentrated. The glue obtained in this way can be made to have the consistency of molasses, and its tenacity is said to be twice that dissolved in hot water. A few pieces of india-rubber of the size of a bullet put into the glue and well shaken will dissolve in a few days, and add to the adhesiveness of the preparation, as well as protect it from the action of moisture.

---

To Make a Good Mucilage.—The best quality of mucilage in the market is made by dissolving clear glue in equal volumes of water and strong vinegar, and adding one fourth of an equal volume of alcohol, and a small quantity of a solution of alum in water. The action of the vinegar is due to the acetic acid which it contains. This prevents the glue from gelatinizing by cooling; but the same result may be accomplished by adding a small quantity of nitric acid. Some of the preparations offered for sale are merely boiled starch, or flour, mixed with nitric acid to prevent the glatinizing.

To Remove Ink Spots from Colored Goods.—An excellent authority states that for fabrics upon which oxalic acid, chloride of lime, and the like cannot be used, a concentrated solution of *pyrophosphate of soda* answers very well. The spot is to be washed with this until it comes out. It requires some patience, especially if the stains to be removed are old ones.

---

A New Winter Salad.—The same excellent authority gives the following: "Ordinary buckwheat, grown in a moderately warm greenhouse, and cut like mustard when about two or three inches high, makes a delicious winter salad. It can be grown in pans all the year round without the least trouble, and even when lettuces are plentiful will be found a very desirable addition to the salad bowl."

---

To Restore Obliterated Writing on Parchment Deeds.—Dip the parchment into a vessel of fresh-drawn spring water, and let it remain about a minute, then take it out, and press it between two sheets of blotting-paper, to prevent its crumbling. When it is nearly dry, examine it, and if the writing is not restored, repeat the operation two or three times. If the fading is only the effect of time, you will, by this means, restore the writing to its pristine state; but if the ink has been removed by any chemical process, of course it cannot be restored.

---

A New Water-Proof Elastic Varnish.—A cheap varnish, capable of withstanding great heat without blistering, and at the same time sufficiently elastic to be applied to fabrics in rendering them water-proof, can be prepared from soap, alum, and turpentine, in the following manner :—

Good yellow soap, containing considerable rosin, is dissolved in boiling water, and to the hot dilute solution alum (or the sulphate of alumina) is added as long as a white alumina soap continues to be formed. This alumina soap is next washed with hot water to remove the adhering salt solution, and then heated to remove the water combined with it. When dried it is as transparent as glycerine soap, and soluble in all proportions in warm spirits of turpentine. Enough of this soap is dissolved in turpentine to make the varnish as thick as damarlack. When used it dries slowly at the temperature of the air, but if the object varnished be exposed to a heat of 112° F., it dries quite rapidly.—*American Artisan.*

GINGER WINE.—This is an excellent stomachic, and is very popular in England as a cheap substitute for grape wine:—

| | | |
|---|---|---|
| Take Sugar | . . . | 12 pounds. |
| Water | . . . . | 3½ galls. |
| Ginger | . . . . | 4 ounces. |

Boil them together for half an hour; when cooled to 75 degrees, add the rinds of six lemons and some good yeast; let it ferment for ten or fourteen days, then add a pint of brandy and bottle it for use.

---

FRECKLES.—For the benefit of young persons afflicted with freckles, we would inform them that powdered nitre, moistened with water, applied to the face night and morning, will soon remove all traces of them; or

Take of

| | |
|---|---|
| Citrine ointment, and | |
| Oil of Almonds of each . . | 1 drachm. |
| Spermatic ointment . . | ¾ of an ounce. |
| Oil of Roses . . . . . . . . | 3 drops. |

Mix well in a wedgwood mortar, using a wooden or bone knife.

---

TO CLEAN GILT JEWELRY.—Take half a pint of boiling water, or a little less, and put it into a clean oil flask. To this add one ounce of cyanide of potassium, shake the flask, and the cyanide will dissolve. When the liquid is cold, add half a fluid ounce of liquor ammonia and one fluid ounce of rectified alcohol.—Shake the mixture together, and it will be ready for use. Gilt articles which have become discolored, may be rendered bright by brushing them with the above mentioned fluid. It must be borne in mind that the cyanide of potassium is a deadly poison, and should be used with caution.

---

TO REPAIR THE SILVERING ON A MIRROR.—The following method has been recommended by good authorities: Clean the bare portion of the glass by rubbing it gently with fine cotton, taking care to remove all dust and grease. With the point of a knife cut upon the back of another glass a portion of the silvering of the required form, but a little larger. Place a drop of mercury upon it. This spreads, and presently the piece of amalgam may be lifted up with the blade of a knife, and transferred to the place to be repaired. Press it with a piece of cotton wool. It hardens speedily, and the patch is as good as any other part of the mirror.

TO CRYSTALIZE FLOWERS.—Construct some basket of fancy form with pliable copper wire, and wrap them with gauze. Into these tie to the bottom violets, ferns, geranium leaves—in fact, any flowers except full-blown roses—and sink them in a solution of alum, of one pound to a gallon of water, after the solution has cooled. The colors will then be preserved in their original beauty, and the crystalized alum will hold faster than when from a hot solution. When you have a light covering of crystals that completely covers the article, remove the basket carefully, and allow it to drip for twelve hours. These baskets make a beautiful parlor ornament, and for a long time preserve the freshness of the flowers.

---

A PERFECT WATERPROOF.—A writer in an English paper says: "By the way, speaking of waterproofs, I think I can give travelers a valuable hint or two. For many years I have worn india-rubber waterproofs, but will buy no more, for I have learned that good Scottish tweed can be made entirely impervious to rain, and, moreover, I have learned how to make it so; and, for the benefit of your readers, I will give the recipe:—

In a bucket of soft water, put half a pound of sugar of lead, and half a pound of powdered alum; stir this at intervals until it becomes clear, then pour it off into another bucket, and put the garment therein, and let it be in for twenty-four hours, and then hang it up to dry without wringing it. Two of my party—a lady and gentleman—have worn garments thus treated in the wildest storms of wind and rain, without getting wet. The rain hangs upon the cloth in globules. In short, they were really waterproof. The gentleman, a fortnight ago, walked nine miles in a storm of rain and wind, such as you rarely see in the South; and, when he slipped off his overcoat, his underclothes were as dry as when he put them on. This is, I think, a secret worth knowing; for cloth, if it can be made to keep out wet, is, in every way, better than what we know as most waterproofs."

---

TO COLLECT THE ODORS OF FLOWERS.—Roses, and all flowers containing perfumed oils, may be made to yield their aromatic properties by steeping the petals of flower leave on a saucer or a flat dish of water, and setting it in the sun. The petals should be entirely covered with the water, which, by the way, should be soft or rain water. A sufficient quantity should be allowed for evaporation, and the vessel should be left undisturbed a few days. At the end of this time a film will be found floating on the top. This is the essential oil of the flower, and every particle of it is impregnated with the odor peculiar to the flower. It should be taken up carefully and put in tiny vials, which should be allowed to remain open till all watery particles are evaporated. A very small portion of this will perfume glove-boxes, drawers, apparel, etc., and will last a long time.

---

Root Beer.—This popular beverage, the season for which is now rapidly approaching, is most satisfactorily made by a process similar to that used for soda water. If the old method of fermentation be adopted, it requires that a number of receptacles for it be in constant use; and the root beer is only in the right condition for use during a limited time, and if kept on hand too long, it runs into the acetic fermentation and gets sour. By the other method it can quickly be made, is ready for use at once, remains good until it is drawn off, and does not sour. We give the following process, employed with great satisfaction by one of the correspondents of the *Druggist's Circular*:—

Sugar house syrup, or nice molasses ½ gal.
Knapp's extract for root beer 1 ounce.
Caramel (to color) 1 to 2 ounces.
*Flavor for root beer 1 ounce.

This may be kept ready mixed in a bottle, and, when necessary, is put into a soda water fountain, with four and a half gallons of water, and then charged with carbonic acid to a pressure of 80 or 100 pounds on the square inch, as indicated by the usual gauge upon the apparatus. Those of our readers having a soda water apparatus can readily use one or more fountains for this purpose with both convenience and profit.

*Flavor for root beer:—
Oil wintergreen 4 drachms.
Oil sassafrass 2 drachms.
Oil cloves 1 drachm.
Alcohol 4 ounces.
Mix.

---

Cold Ablutions in Fever.—In an article contributed to a French medical journal, Dr. Lambert arrives at the following conclusions concerning the use of cold ablutions in fever:—

1. They are especially useful in typhoid and the eruptive fevers, and strongly indicated in malignant cases.

2. They act upon the chief and most constant phenomena of these diseases, and are especially antifebrile, reducing the temperature 1.5° to 3° Cent.

3. They favor the re-establishment of a full, profound, regular respiration.

4. They favor the peripheral circulation by the strong rhythmical contractions of the small vessels, which they produce in a reflex manner.

5. They render the secretions more active.

6. They make the skin supple, moist, an fresh.

7. They favor the outcoming of the eruption.

8. They calm cerebral and other nervous excitement, suppressing headache, coma, delirium, restlessness, and causing sleep.

9. They cause the pulse to fall 8 to 30 beats.

10. Their action persists from two to eight hours, and they ought to be repeated two to four times in the twenty-four hours.

11. They have no influence upon the length of the sickness, but render it milder.

12. They are not disagreeable; are readily applied, either as cold baths, or by rapping the patient in a cold wet sheet.

---

Splendid Poster.—The Pennsylvania Railroad Company have just issued a large poster representing Watkins Glen and Rainbow Falls. It is printed in six different colors, and is a splendid specimen of color printing.

---

Smith and Jones were in a menagerie, and the conversation turned on Darwin's theory. "Look at that monkey," said Smith. "Think of its being an undeveloped human!" "Human!" said Jones, contemptuously. "It's no more human than I am."

---

"Salts of demoniac" were recently called for at a country store in Western Massachusetts. The apothecary filled the bill with a pint of New England rum. Anything more demoniac than that, he said, wasn't down in his materia medica.

